# Antioxidant and Anti-Inflammatory Activities of Extracts from *Cassia alata, Eleusine indica, Eremomastax speciosa, Carica papaya* and *Polyscias fulva* Medicinal Plants Collected in Cameroon

**DOI:** 10.1371/journal.pone.0103999

**Published:** 2014-08-04

**Authors:** Bertrand Sagnia, Donatella Fedeli, Rita Casetti, Carla Montesano, Giancarlo Falcioni, Vittorio Colizzi

**Affiliations:** 1 Laboratory of Microbiology and Immunology, Chantal BIYA International Reference Centre for Research on Prevention and Management of HIV/AIDS (CIRCB), Yaounde, Cameroon; 2 School of Pharmacy, University of Camerino, Camerino (MC), Italy; 3 Laboratory of Cellular Immunology, National Institute for Infectious Diseases “Lazzaro Spallanzani”, Rome, Italy; 4 Department of Biology, University of Rome Tor Vergata, Rome, Italy; University of Sevilla, Spain

## Abstract

**Background:**

The vast majority of the population around the world has always used medicinal plants as first source of health care to fight infectious and non infectious diseases. Most of these medicinal plants may have scientific evidence to be considered in general practice.

**Objective:**

The aim of this work was to investigate the antioxidant capacities and anti-inflammatory activities of ethanol extracts of leaves of *Cassia alata, Eleusine indica, Carica papaya, Eremomastax speciosa* and the stem bark of *Polyscias fulva*, collected in Cameroon.

**Methods:**

Chemiluminescence was used to analyze the antioxidant activities of plant extracts against hydrogen peroxide or superoxide anion. Comet assays were used to analyze the protection against antioxidant-induced DNA damage induced in white blood cells after treating with hydrogen peroxide. Flow cytometry was used to measure γδ T cells proliferation and anti-inflammatory activity of γδ T cells and of immature dendritic cells (imDC) in the presence of different concentrations of plant extracts.

**Results:**

Ethanol extracts showed strong antioxidant properties against both hydrogen peroxide and superoxide anion. *Cassia alata* showed the highest antioxidant activity. The effect of plant extracts on γδ T cells and imDC was evidenced by the dose dependent reduction in TNF-α production in the presence of *Cassia alata*, *Carica papaya, Eremomastax speciosa Eleusine indica,* and *Polyscias fulva*. γδ T cells proliferation was affected to the greatest extent by *Polyscias fulva*.

**Conclusion:**

These results clearly show the antioxidant capacity and anti-inflammatory activities of plant extracts collected in Cameroon. These properties of leaves and stem bark extracts may contribute to the value for these plants in traditional medicine and in general medical practice.

## Introduction

Medicinal plants have been used in traditional health care systems since prehistoric times and are still the most important health care source for the most of the world’s population. The World Health Organization (WHO) has estimated that more than 75% of the world’s total population depends on herbal drugs for their primary healthcare needs [1]. Therefore, there is a major research emphasis on discovering plants with antioxidant and anti-inflammatory potential that may be treat various kinds of injuries or protect against diseases [2]. Cellular and tissue damage caused by oxidative stress is defined by the elevated levels of free radicals or other reactive oxygen species (ROS) that can elicit direct or indirect damage to the body. The generation and subsequent involvement of free radicals contributes to a large number of diseases including AIDS [3], [4], carcinogenesis and liver damage, inflammatory diseases, cataract formation and Alzheimer’s disease, are recognized [5], [6], [7], [8]. Intracellular protective mechanisms against inflammatory stresses involve antioxidant enzymes, including superoxide dismutase (SOD), catalase (CAT) and glutathione peroxidase (GPx) in tissues. It has been shown that faulty cellular antioxidant systems cause organisms to develop a series of inflammatory or malignant diseases [9]. However, it appears that the various roles of enzymatic antioxidants help to protect organisms from excessive generation of oxidative stress during inflammation process, which leads to studies focusing on the role for natural products in suppressing the production of oxidation by increasing enzymatic antioxidants in tissues [10].

Under normal conditions, ROS levels are controlled by the body’s complex antioxidant defense system and there is an equilibrium between ROS formation and degradation. Overproduction of ROS and/or inadequate antioxidant defense disturbs this equilibrium in favor of a ROS upsurge that results in oxidative stress. A deficiency in the body’s natural antioxidant defense mechanisms has been implicated as the etiological or pathological factor in several clinical disorders. This has led to scientific research focusing on identifying safe and effective sources of antioxidants. Plant extracts and plant-derived antioxidant compounds may potentiate the body’s antioxidant and anti-inflammatory defense mechanisms or act as antioxidants.

Inflammation is a normal response to tissue injury but, if uncontrolled, leads to additional complications. At the injury site, an increase in blood vessel wall permeability followed by migration of immune cells can cause edema formation during inflammation. Excessive inflammation contributes to many acute and chronic human diseases [11].

The immune system continuously monitors resident microbiota and utilizes constitutive antimicrobial mechanisms to maintain immune homeostasis. For example, lipopolysaccharide (LPS) is an endotoxin and a constituent of the outer membrane of gram-negative bacteria. LPS stimulates innate immunity, by regulating the productions of inflammatory mediators, like, Nitric Oxide (NO), TNF-α (Tumor Necrosis Factor-alpha), Interleukin-6 (IL-6), prostanoids, and leukotrienes. Dendritic cells (DCs), the main antigen presenting cells, play a pivotal role in priming T cells upon immune challenge [12]. DCs respond to innate immune activation by upregulating costimulatory molecules, lymphoid-homing chemokines and producing proinflammatory cytokines, such as TNF-α. There is a reciprocal activation between DCs and Vγ9Vδ2 T cells, which are the major population of peripheral blood γδ T cells [13], [14]. Vγ9Vδ2 T cells, when activated by phosphoantigen compounds after viral or bacterial infection, produce some cytokines and chemokines that recruit other immune cells to the site of infection. TNF-α is one of the most pro-inflammatory cytokine produce by Vγ9Vδ2 T cells when activated [15] and if this reaction is not controlled, it may lead to chronic, destructive inflammation. Thus, antioxidant and anti-inflammatory activities are very important for controlling oxidative stress and inflammatory processes generated during the response to infectious diseases.

In this study, we used plant extracts of *Cassia alata* Linn (Caesalpinaceae), *Carica papaya* Linn (Caricaceae), *Eremomastax speciosa* Hochst (Acanthaceae), *Eleusine indica* Linn Gaertn (Poaceae) and the stem bark of *Polyscias fulva* Hiern HARMS (Araliaceae) to evaluate their antioxidant activities compared to the antioxidant enzyme present in humans, the superoxide dismutase (SOD).

Leaves of *Carica papaya*, L. [16], have been used to treat malaria, yellow fever, oral candidosis [17], dengue, as a diuretic and in case of anemia [18], [19], [20], [21]. Leaves of *Eremomastax speciosa* Hochst, are used as anti-diarrheal [22], [23]. Leaves of *Cassia alata* are used against yellow fever or malaria, and as anti asthmatics, or anti diabetics [24], [25], [26]. *Eleusine indica* Gaertn or Wiregrass (grass family Poaceae), is used in traditional medicine as a diuretic, anti-helminthic, febrifuge and for treating cough [27], [28]. *Eleusine indica* was evaluate for hepatoprotective effects and its mechanism of action was studied [29]. *Polyscias fulva,* Harms, is used in traditional medicine to fight against venereal infections and against dermatoses [30], [31]. Since these plants are widely used in Cameroon as traditional folk medicines there is a need for chemical and pharmacological studies that may help to corroborate the scientific bases of the use of these plant products and standardize their use in general medical practice. The *E. coli* plasmid, the DNA of lymphocytes and the liposomes were used as a model to evaluate the antioxidant properties of plant extracts against anion superoxide and hydrogen peroxide. γδ T cells and imDC were used as a model to evaluate the anti-inflammatory activity. Our results showed that certain medicinal plants are promising sources of potential antioxidants and anti-inflammatory and may be effective as preventive agents in the pathogenesis of some diseases.

## Materials and Methods

### Plant Material

Fresh aerial parts of *Carica papaya* (leaves), *Eremomastax speciosa* (leaves), *Eleusine indica* (leaves), *Cassia alata* (leaves), and *Polyscias fulva* (stem bark), were collected in the Centre Region of Cameroon, in January–June, 2002. These plants were identified at the Cameroon National Herbarium, where the voucher specimens were kept under the reference numbers.

### Ethics Statement

For the collection of plants, no specific permits were required for the described field studies. For any locations/activities, no specific permissions were required. All locations where the plants were collected were not privately-owned or protected in any way and the field studies did not involve endangered or protected species. This study was approved by the University of Camerino’s institutional review board.

### Extraction

Air-dried and powdered samples from each plant were macerated separately in an ethanol for 48 h at room temperature with shaking. The extract was filtered through a Whatman no. 1 filter paper. The filtrate was then evaporated to dryness at 50°C for ethanol under reduced pressure using a rotary evaporator. This produced a residue which constituted the crude extract. The extraction yield was calculated and the crude extract was kept at +4°C until further use.

### Chemiluminescence Measurement of Plant Extracts Antioxidant Activity

Chemiluminescence measurements (CL) to evaluate the antioxidant activity of plant extracts were performed using an Autolumat Berthold LB 953 (Berthold Co., Wilbard, Germany). In order to measure the scavenger activity of these compounds against hydrogen peroxide, a reaction mixture containing different concentrations of plant extracts, 100 µM luminol in 50 mM phosphate buffer pH 7.0 were prepared. The reaction was initiated by injecting 0.05 ml of H_2_O_2_ to a final concentration of 50 mM. To assess the scavenger activity toward superoxide anion, a reaction mixture containing 0.9 U/ml Xanthine oxidase, 150 µM lucigenin in 50 mM phosphate buffer pH 7.0, and different concentrations of plant extracts were used. The reaction was started by injecting Xanthine at a final concentration of 50 µM. The data were reported as the percentage (%) of inhibition (I) of the CL (chemiluminescence) signal and calculated as follows:

where ***CL*** **sample** is the chemiluminescence signal obtained for a sample in the presence of plant extracts and ***CL*** **blank** is the chemiluminescence signal obtained in the sample without plant extracts.

### *E. coli* DNA damage

The antioxidant capacity of plant extracts was evaluated on *E. coli* plasmid DNA pBR322 in the presence of oxidant agents. 100 ηg DNA +10 µM Fe^++^ +100 µM H_2_O_2_+12.5 µg plant extracts. Samples were incubated for 1 h at 35°C. The agarose gel (1.2%) was prepared by dissolving the solid agarose powder in the electrophoresis buffer, the Tris-AceticAcid-EDTA (“TAE”). (A commonly used stock solution for TAE is 50 times concentrated (“50xTAE”); the standard procedure for preparation of 50xTAE is: mix deionised water with solid Tris powder, a certain amount of an EDTA stock solution, usually 0.5 M EDTA pH 8.0, and concentrated acetic acid to adjust the pH to 7.6). The agarose gel was dissolved at 100°C for a few minutes and the dissolved solution was then poured into an electrophoretic plate which contained combs. Cooling down to room temperature results in the slow formation of a solid gel when the concentration of agarose is between 0.5 and 2% (weight/volume). Ethidium bromide (Et-Br) was added to the gel solution for visualization of DNA bands. The Et-Br agarose gel was then submerged in a horizontal electrophoresis apparatus. Loading buffer (Bromo Phenol Blue) was used to fill the samples into the wells and to serve as the migration tracking dye. Migration was done at about 85 Volts for 45 min at room temperature. After electrophoresis, the gel was placed under UV light and standard or digital photograph of the fluorescent DNA bands were saved.

### Comet Assay

DNA damage in lymphocytes was evaluated using the alkaline single-cell microgel electrophoresis (‘comet’ assay) [32], [33]. The comet assay was carried out under yellow light. About 2×10^5^ cells were mixed with 65 µl of 0.7% low melting agarose (LMA) in Ca^2+^ and Mg^2+^ free PBS to form a cell suspension. The cell suspension was rapidly spread over a pre-cleaned microscope slide previously conditioned by spreading a 1 ml aliquot of 1% LMA in Ca^2+^ and Mg^2+^ free PBS. After solidification, the cells were protected with a top layer of 75 µl of 0.7% LMA. To lyse the embedded cells and to permit DNA unfolding, the slides were immersed in freshly prepared ice-cold lysis solution (1% sodium *N*lauroyl- sarcosinate, 2.5 M NaCl, 100 mM Na_2_EDTA, 10 mM Tris–HCl, pH 10, with 1% Triton X-100 and 10% DMSO added just before use) for 1 h at +4°C in the dark. After the lysis, the slides were placed on a horizontal electrophoresis box. The unit was filled with freshly made alkaline buffer (300 mM NaOH, 1 mM Na_2_EDTA, pH>13) and, to allow DNA unwinding and expression of alkali labile damage, the embedded cells were left in the solution for 20 min. Electrophoresis was performed for 20 min by applying an electric field of 25 V and adjusting the current to 200 mA. After the electrophoresis, the slides were washed gently with 0.4 M Tris- HCl buffer pH 7.5 to neutralize the excess alkali and remove detergents. Slides were stained by adding 20 µl of ethidium bromide (2 µg/ml) and the rate of DNA damage was evaluated by analyzing at a magnification of ×20 the images of 150 randomly selected cells (50 cells from each of three replicate slides) by using an Axioskop 2 plus epi-fluorescence microscope (Carl Zeiss, Germany) equipped with an excitation filter of 515–560 nm. Imaging was performed using a specialized analysis system (‘Metasystem’ Altlussheim, Germany) to determine tail length, tail intensity and tail moment.

### Inhibition of lipid peroxidation assay

Fe^2+^ induced lipid peroxidation is one of the established system for assessing antioxidant action of different plant extracts. A modified thiobarbituric acid-reactive species (TBARS) assay [34], [35] was used to measure the lipid peroxide formed using liposomes homogenate as lipid rich media. Malondialdehyde (MDA), a secondary end product of the oxidation of polyunsaturated fatty acids, reacts with two molecules of TBA yielding a pinkish red chromogen, homogenate was centrifuged at 800 g for 15 min at 4°C and the supernatant was used in thiobarbituric acid assays. Different concentrations of plant extracts (40–400 µg/mL) were fixed with the liposome preparation and incubated at room temperature for 10 min. Then, 50 µL Fenton’s reagents (10 mM FeCl3; 10 µL of 2.5 mM H_2_O_2_) in phosphate buffer (0.2 M, pH 7.4) were added, and the volume was made to 1 mL. The tubes were then incubated for 30–45 min at 37°C to induce lipid peroxidation. Thereafter, 2 mL of ice-cold HCl (0.25 N) containing 15% trichloroacetic acid, 0.5% thiobarbituric acid and 0.5% butylated hydroxytoluene (BHT) were added in each sample that were boiled for 15 min. The mixture was then centrifuged at 1000 rpm for 10 min and the extent of lipid peroxidation was subsequently monitored by formation of thiobarbituric acid reactive substances (TBARS) as pink chromogen in presence or absence of extracts. The absorbance of the supernatant was measured spectrophotometrically at 532 nm and decline in formation of pink chromogen in pre-treated reactions was considered as inhibition of lipid peroxidation.

### Cell isolation and culture conditions

PBMC from healthy donors were isolated from fresh buffy coats by Lympholyte (Cedarlane) density gradient centrifugation. Cells were cultured at a concentration of 4×10^6^/ml in the presence of IL-2 (6.5 U/ml), IL-15 (10 ηg/ml) (both from Sigma-Aldrich), TGF-β (1.7 g/ml; Calbiochem) and isopentenyl pyrophosphate (IPP; 20 µg/ml; Sigma). On days 3, 6, and 9, half of the supernatant volume was discarded and replaced with fresh medium containing cytokines. Aliquots of PBMC and bead-sorted *γδ* T cells (Miltenyi Biotec) were used fresh or were frozen for use at later time points.

### Monocyte purification, imDC generation

PBMCs were isolated from buffy coats of healthy donors by density gradient centrifugation using Lympholyte-H (Cederlane Laboratories). In this study we used anonymous residual samples (buffy coats) and approval was not necessary. These residual samples were collected in the Azienda Ospedaliera Universitaria Policlinico Umberto I - Centro Trasfusionale e Immunoematologia. URL: www.policlinicoumberto1.it.

Monocytes were positively separated by anti-CD14 magnetic beads (MACS; Miltenyi Biotec) according to the manufacturer’s instructions. The cells were then resuspended in RPMI 1640 (Euroclone) supplemented with 10% FCS (HyClone, Invitrogen Life Technologies), L-glutamine (2 mM), HEPES buffer (10 mM), and gentamicin (10 µg/ml) (Sigma-Aldrich), and cultured for 5 days in the presence of GM-CSF (200 U/ml) and IL-4 (10 ηg/ml; Euroclone) to generate immature DC (imDC). Then, imDC were put in culture with different concentration of plant extracts in the presence of 200 ηg of LPS.

### *γδ* T cells purification and proliferation

*γδ* T cells were separated from autologous PBMCs by positive selection using anti-*γδ*-magnetic beads (MACS; Miltenyi Biotec) according to the manufacturer’s instructions. Purified cell populations contained 98% of viable *γδ* T cells as assessed by flow cytometry. Purified-*γδ* T cells were incubated for 10 days with 3 µg of IPP and 100 U/mL of Interleukine-2 (IL-2) (Euroclone) and in the presence of different concentrations of plant extracts.

### FACS analysis

The following FITC-, PE-, PerCP-, or APC- conjugated Abs: CD25, CD14, CD27, CD45RA, CD62L, TNF-α, IFN-γ, CD3, HLA-DR, and Vδ2 (BD Biosciences) were used for direct immunofluorescence staining to characterize the phenotype of *γδ* T cells. Isotype-matched mAbs (BD Biosciences) were used in all experiments as controls. In brief, the cells were washed twice in PBS, 1% BSA, and 0.1% sodium azide, and were stained with the mAbs for 15 min at 4°C. The cells were then washed and analyzed using a FACSCalibur instrument with CellQuest software (BD Biosciences). The following PE and allophycocyanin-conjugated anti-TNF-α and anti-IFN-γ mAbs (BD Pharmingen) were used for intracellular immunostaining to characterize Vδ2 T cells and imDC producing cytokines.

### Statistical analysis

Statistical analysis was determined using a Mann-Whitney *U* test. Values of *p*<0.05 were considered statistically significant for flow cytometry. Data are expressed as mean values SEM except for chemiluminescence results, for which data are presented as mean values SD.

## Results

In this study we evaluated both antioxidant and anti-inflammatory activity of *Cassia alata* Linn (Caesalpinaceae), *Carica papaya* Linn (Caricaceae), *Eremomastax speciosa* Hochst (Acanthaceae), *Eleusine indica* Linn Gaertn (Poaceae) and the stem bark of *Polyscias fulva* Hiern HARMS (Araliaceae) by using chemiluminescence, gel electrophoresis and flow cytometry respectively. First, we showed the presence of phenol in all extracts by the spectrophotometric analysis.

### Antioxidant scavenging activities

**Figure 1:**
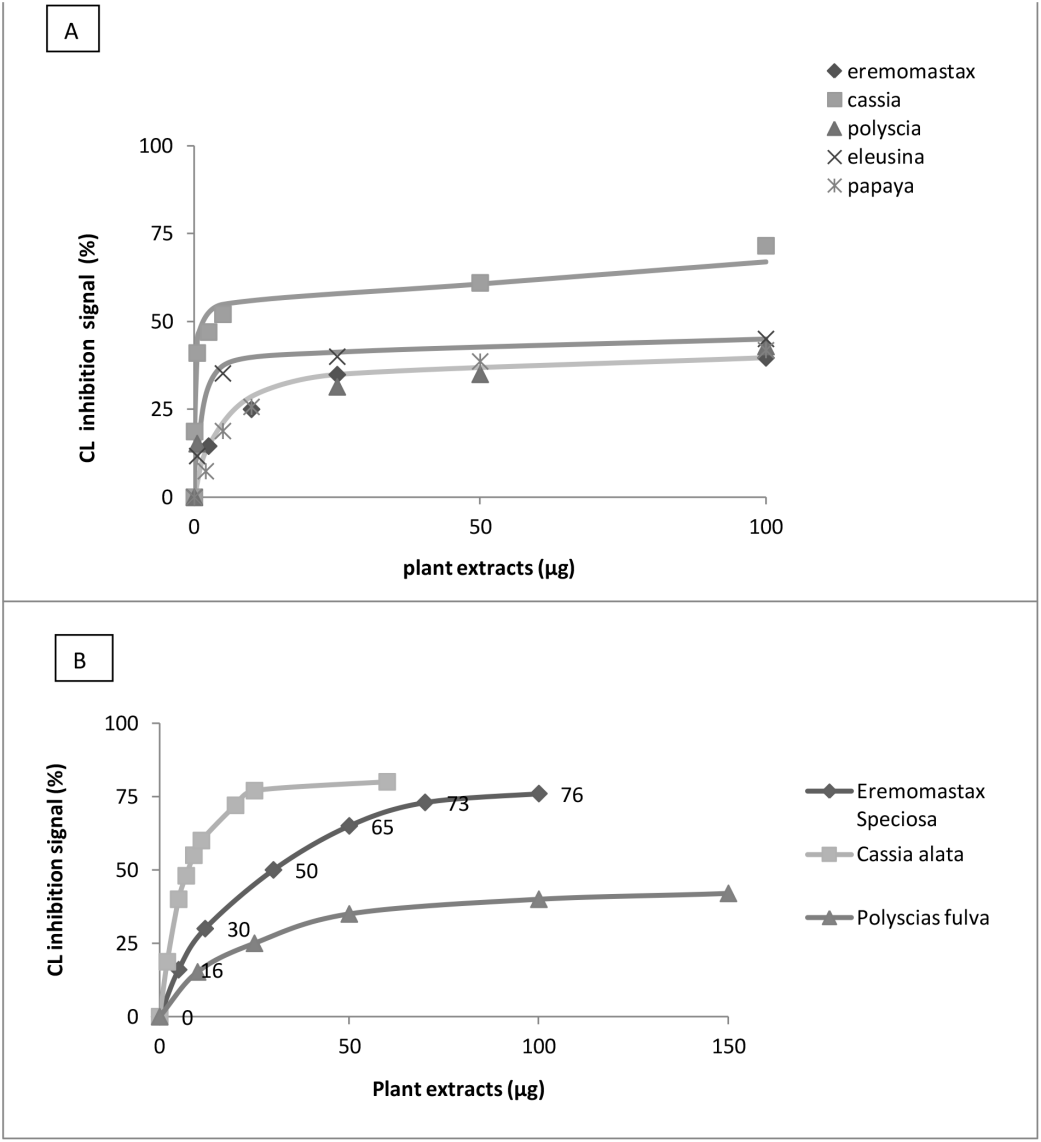
Evaluation of the chemiluminescence (CL) signal inhibition in presence of different concentrations of plant extracts versus: A) Hydrogen peroxide (H_2_O_2_) B) Anion superoxide (O_2_) generated by Xanthine – Xanthine oxidase system.

**Figure 2:**
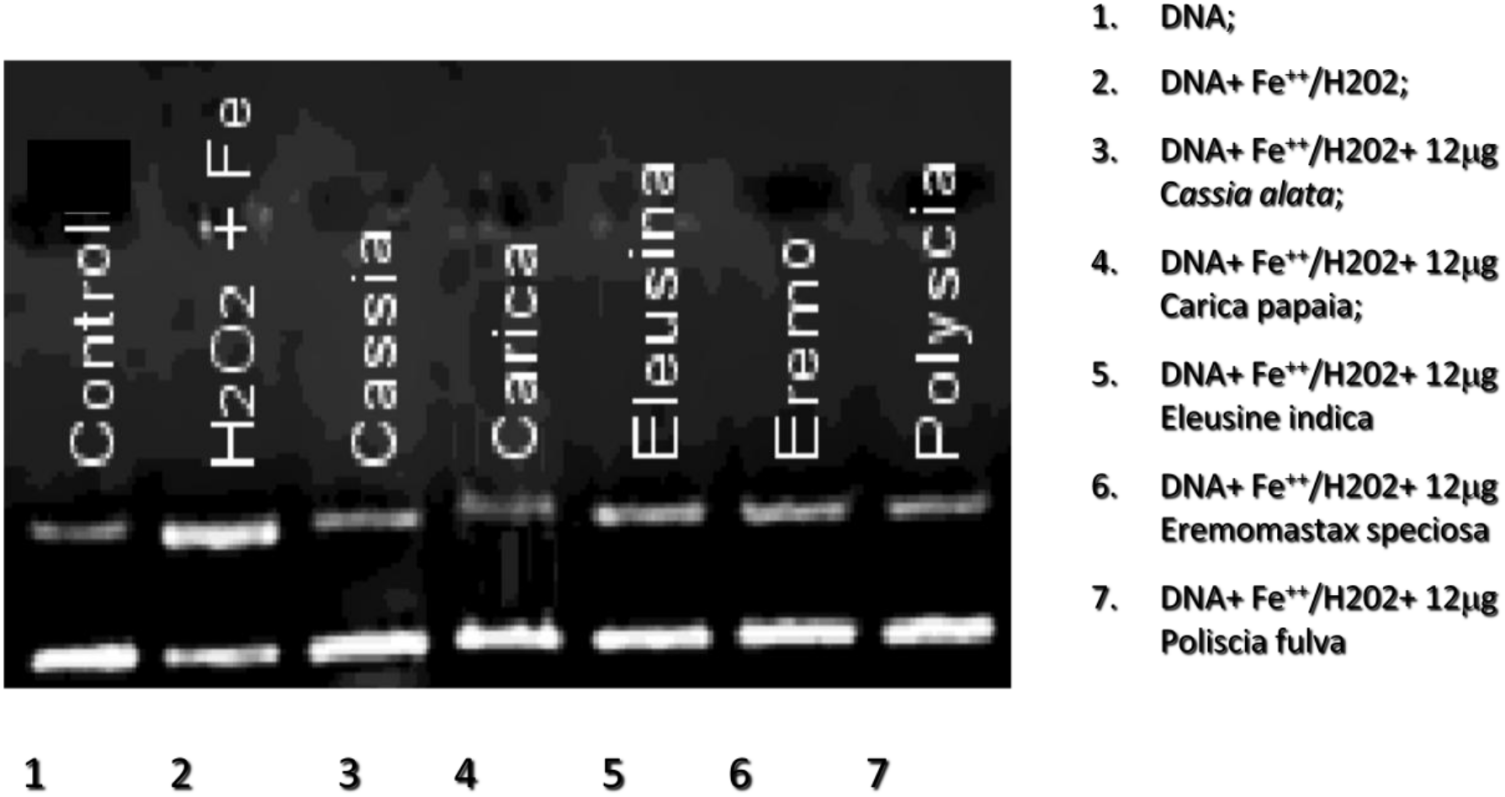
Protective effect of 12 µg plant extracts from Fe^2+^ and H_2_O_2_ induced *E. coli* plasmid damage. *Ctr: control.*

In order to evaluate the scavenger activities of plant extracts against hydrogen peroxide or superoxide anion, luminol and lucigenin amplified chemiluminescence inhibition respectively was measured. As showed in Fig. 1A, Luminol amplified chemiluminescence signal reduced of 67%, 45.5%, 42.9%, 41.8% and 39.7% for *Cassia alata, Eleusine indica, Polyscias fulva, Carica papaya* and *Eremomastax speciosa*, respectively by using less than 12.5 µg of plant extracts. Concerning the scavenger activity versus superoxide anion generated by xanthine-xanthine oxidase system, lucigenin amplified chemiluminescence was reduced of 65%, 38% and 10% in presence of *Cassia alata, Eremomastax speciosa* or *Polyscias fulva*, respectively Fig. 1B. For both systems, *Cassia alata* resulted to have the best scavenger activity. Moreover, the antioxidant capacity of plant extracts was evaluated by measuring the reduction of Fe^3+^ ferricyanide complex to the ferrous form, Fe^2+^ due to the presence of a reductant, such as the antioxidant substances in plant extracts. As shown in Fig. 2, 12 µg of plant extracts, protect the E. coli plasmid from the oxidation with hydrogen peroxide by maintaining the DNA plasmid supercoiled. The relevant information is conversion of supercoiled DNA to the slower running nicked circular DNA. The protective effects of plant extracts are seen by the reduction in nicked circular DNA.

**Figure 3:**
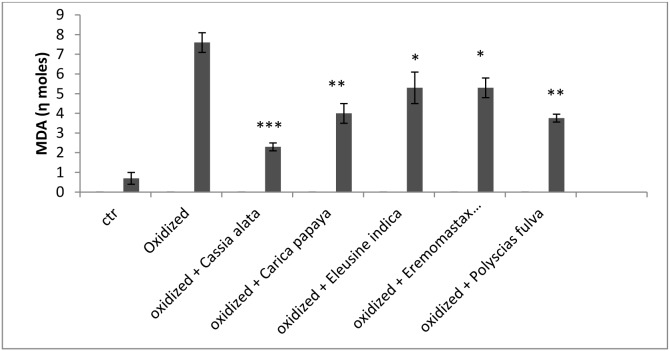
Reduced amount of malonil dialdeide (MDA) by lipid peroxydation in presence of a lucegenin probe and the xanthine/xanthine oxydase system with 150 µg of different plant extracts. Ctr: Control, ***p<0.001, **p<0.01, *P<0.05.

In Fig. 3, the plant extracts were tested on chicken liposomes about the reduction of MDA (Malonil dialdeid) in presence of the anion superoxide produced by Xanthine – Xanthine oxidase and amplified by lucegenin as a probe. In this system, using 150 µg of plant extracts, the highest reducing power activity was observed on *Cassia alata*, followed by *Polyscias fulva, Carica papaya, Eremomastax* and *Eleusine indica*, if compared with the positive control. The ferric reducing power may be attributed to the phenolic and flavonoid contents of the extracts. The ability to reduce Fe (III) may be attributed to the hydrogen donation from phenolic compound, which is related to the presence of a reducing agent. In addition, the number and position of hydroxyl group of phenolic compounds also govern their antioxidant activity [36].

**Figure 4:**
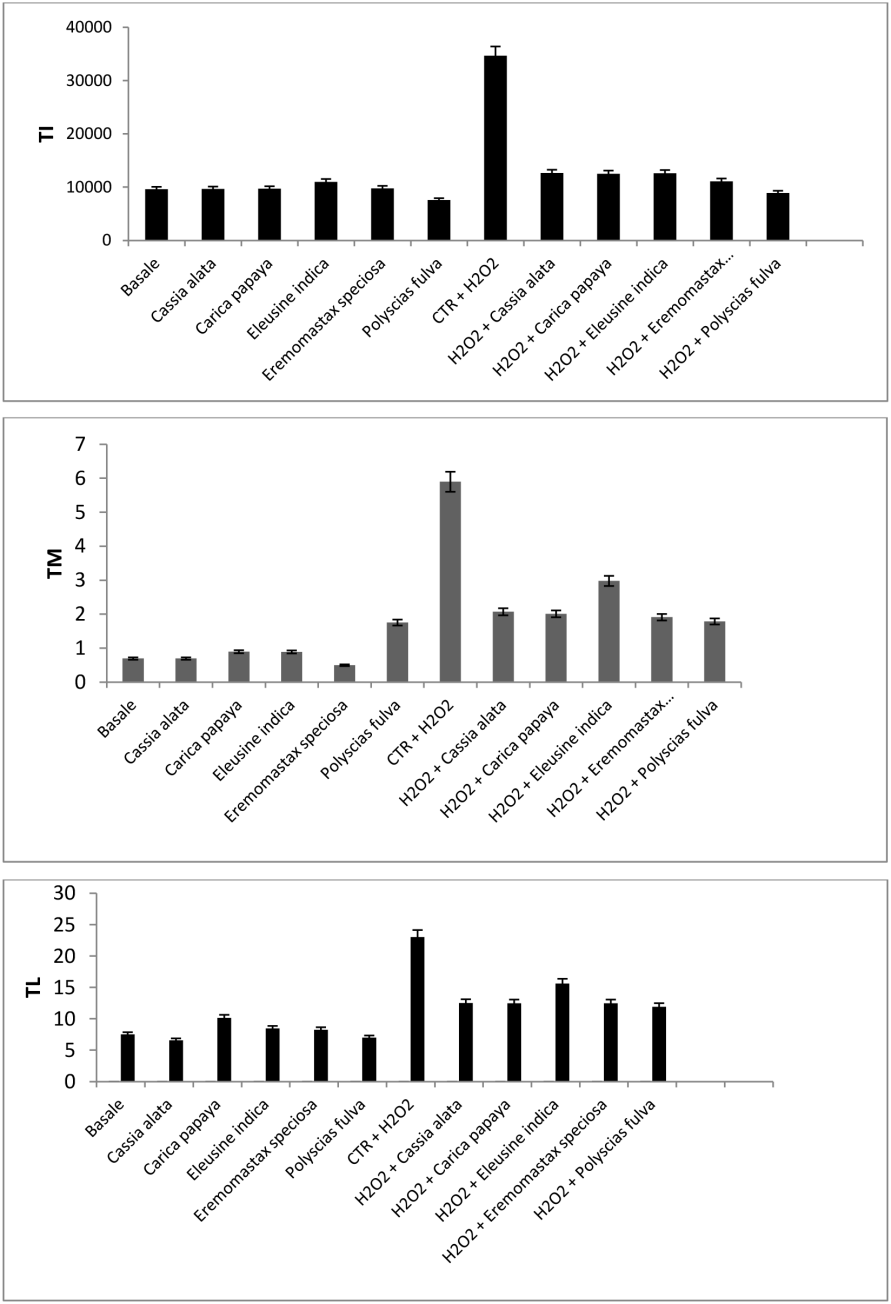
Distributions of: a) comet tail moment (TM); b) tail length (TL); c) tail intensity (TI) in white blood cells from healthy human donors treated with hydrogen peroxide and 100 µg of plant extracts. Data (at least 150 scores/sample) are mean values +/− SEM. *p*<0.05 is considered significant.

For evaluating DNA damage in lymphocytes following hydrogen peroxide treatment and to explore the potential protective effect of plant extracts the comet assay was used. Comet assay parameters – tail moment, tail length, and tail intensity – have been used widely all over the world for determination of DNA damage. As the amount of the damage increases in a cell, more DNA migrates into the tail intensity and is quantified in terms of increased amount of determined fluorescence in the tail region and tail length. The percentage of DNA in the tail region (tail intensity) has been in use for quantifying DNA strand breakage and is the most advised parameter to use [37], [38]. A major advantage of using tail moment as the index of DNA damage is that both the amount of damaged DNA and the distance of migration of the genetic material in the tail are represented by a single number. In this study, data analysis was performed using tail moment, tail length, and tail intensity. In our study, the main outcome of the comet assay was the detection of DNA strand breaks caused by exposure to hydrogen peroxide. Fig. 4 shows the data obtained after incubating cells with 100 µg of plant extracts in the presence or absence of hydrogen peroxide. Our results show that plant extracts protect the DNA of lymphocytes from damage because there was a significant decreased of Tail Intensity (Fig. 4A), Tail Moment (Fig. 4B), and Tail length (Fig. 4C) in the Comet assays.

### Anti-inflammatory activity

As known, Lipopolysaccharide (LPS) induce dendritic cell maturation producing a great amount of TNF-α. To assess the anti-inflammatory activity of plant extracts, TNF-α production by imDC was evaluated after culture of DC with different concentration of plant extracts in the presence of LPS.

**Figure 5:**
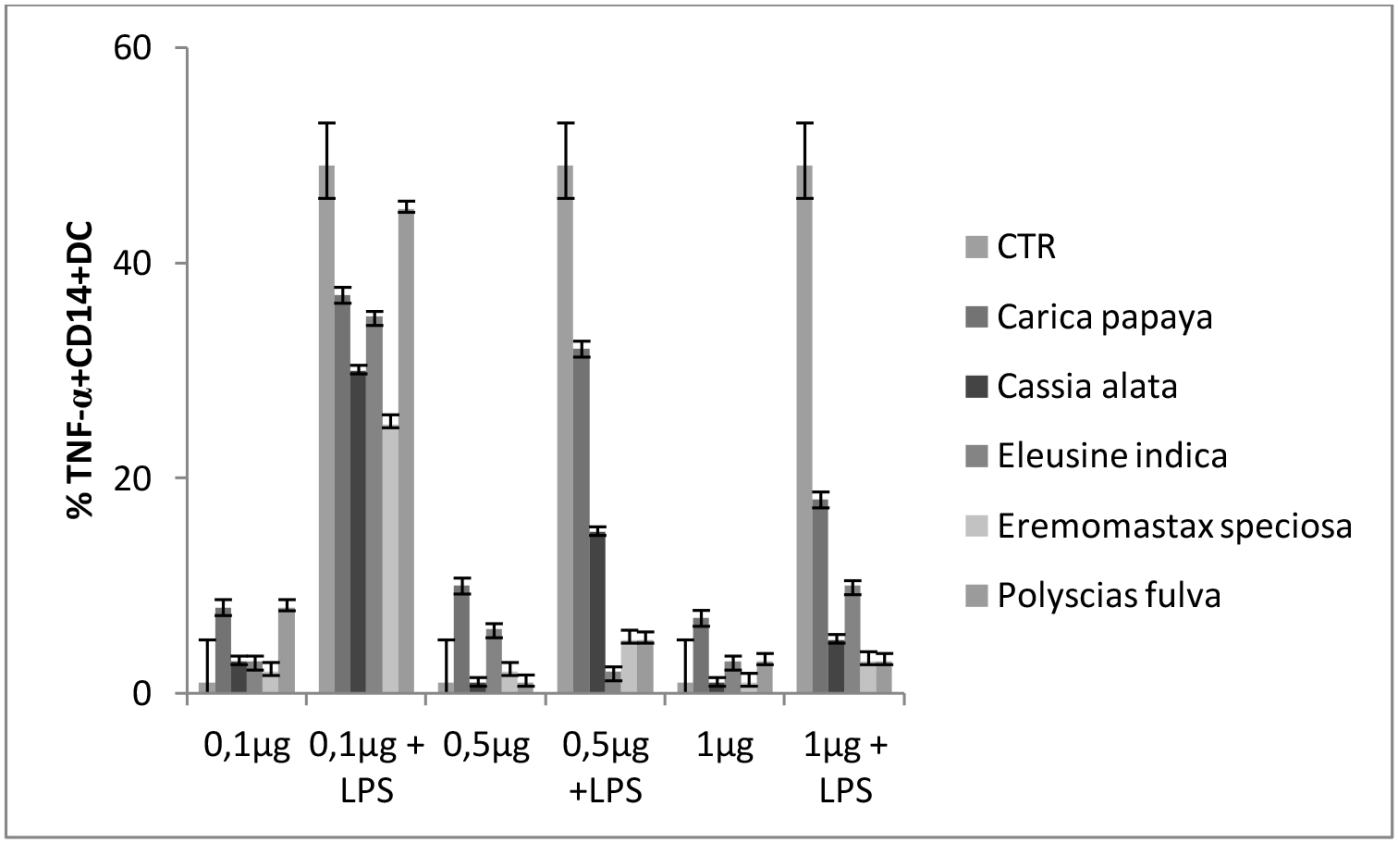
Plant extracts modulate LPS –induced TNF-α production by imDC. imDC were generated from monocytes positively separated from PMBC by anti-CD14 magnetic beads and cultured for 5 days in the presence of GM-CSF and IL-4. Different concentrations of plant extracts were added to imDC in the presence of LPS and TNF-α production was assessed by Flow Cytometry. Values of *p*<0.05 were considered statistically significant. *CTR: Control; imDC: immature Dendritic Cells; LPS: Lipopolysaccharide; TNF-α: Tumor necrosis factor alpha.*

As shown in Fig. 5, all the considered plant extracts inhibited in a dose dependent manner the LPS-induced TNF-α production by imDC. The most inhibition was obtained with 1 µg of plants extracts by *Eremomastax speciosa Polyscias fulva* and *Cassia alata* followed by *Eleusine indica* and *Carica papaya.*

**Figure 6:**
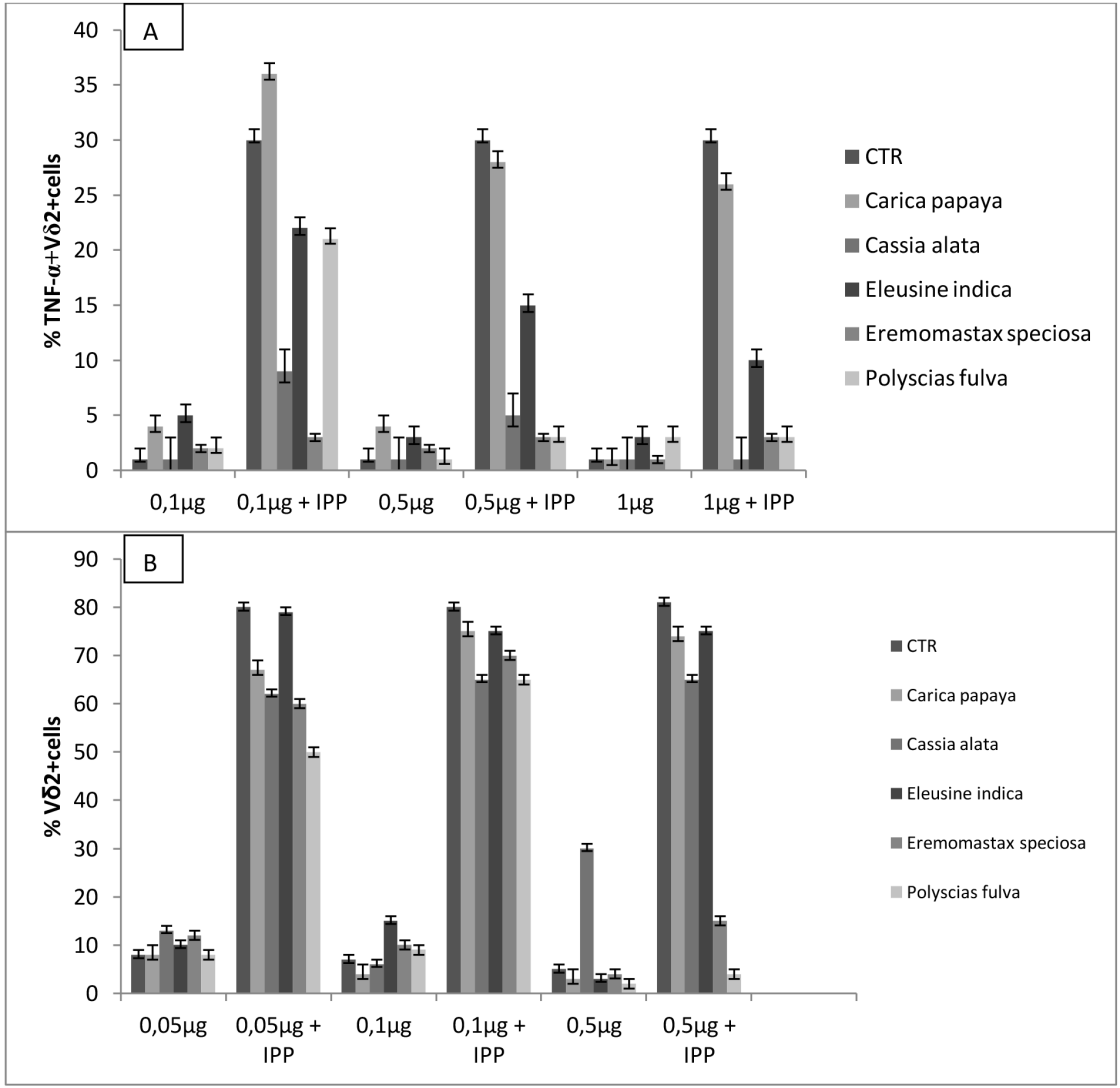
Plant extracts modulate γδ T cell cytokine production and proliferation. Purified-*γδ* T cells were incubated with IPP and IL-2 in the presence of different concentrations of plant extracts induced TNF-α production (A) and on γδ T cell proliferation (B) were assessed by Flow Cytometry. Values of *p*<0.05 were considered statistically significant. *CTR: Control; TNF-a: Tumor necrosis Factor-alpha; IPP: isopentenyl pyrophosphate.*

In response to phosphoantigen stimulation γδ T cells produce high levels of cytokines including TNF-α and begin to proliferate. In our experiment, we evaluated the effect of plant extracts on both the cytokine production and proliferation of γδ T lymphocytes. IPP-induced TNF-α production was inhibited by plant extracts in a dose dependent manner. The greatest inhibition was obtained by 1 µg of *Cassia alata*, followed in the same extent by *Eremomastax speciosa* and *Polyscias fulva,* and finally by *Eleusine indica* and *Carica papaya* (p<0.05) (Fig. 6A).

As regards the proliferative activities, PBMCs of healthy donors were incubated for 10 days with IPP and different concentrations of plant extracts in the presence of IL-2. As shown in Fig. 6B, only *Eremomastax speciosa* and *Polyscias fulva* at 0.5 µg inhibited γδ T cells proliferation.

Taken together these results show that *Cassia alata*, but also *Eremomastax speciosa* and *Polyscias fulva* possess the best antioxidant and scavenger activities as well as anti-inflammatory activity.

## Discussion

In this study, we used plants extracts to evaluate their antioxidant activities compared to the antioxidant enzyme present in humans, the superoxide dismutase (SOD). The *E. coli* plasmid, the DNA of lymphocytes and the liposomes were used as a model to evaluate the antioxidant properties of plant extracts against anion superoxide and hydrogen peroxide. γδ T cells and imDC were used as a model to evaluate the anti-inflammatory activity. The antioxidant and anti-inflammatory activities are very important for humans because the oxidative stress and the inflammatory process were generated during different infectious diseases. In this study, we observed that extracts from leaves of *Cassia alata* as well as *Eremomastax speciosa*, and *Polyscias fulva* have better antioxidant activities than *Carica papaya* or *Eleusine Indica*. This could be explained in part because the spectrophotometric analysis show the presence of phenols in all extracts and these extracts protect from hemolysis except for the *Polyscias fulva* stem bark extract. The antioxidant and anti-inflammatory activities could also been explained by the chemical analysis of *Cassia alata*, showing the presence of the Chrysarobin, the tannin, Kaempferol, Isochrysophanol, Chrysophanol glycoside Chrysarobin 1,5 [24]. Leaves of *Eremomastax speciosa* show the presence of alkaloids, of iridoids, and arthraquinone, flavonols like kaempferol and trace of quercetin [39]. Chemical analyses of the sterm bark of *Polyscias fulva* present the tri terpène glycoside drammarane-type, 2 types of saponin (α -hederin and 3-O- α -L-rhamnopyranosyl-(1→2)- α -L-arabinopyranosyl-hederagenin-28-O-α-L-rhamnopyranosyl-(1→4)-β-.glucopyranosyl-(1→6)- β-D-glucopyranosyl ester and the quercetin 3-O-β-D-glucopyranoside [31]. Leaves of *Carica papaya* are rich in anthraquinone and in alkaloids like carpaine, the flavonols, the vitamin C and E. These molecules have a wide range of biological activities. The popularity of herbs in traditional medicine has been linked to their higher likelihood of containing pharmacologically active compounds compared to woody plant forms [40]. Previous studies showed chemical constituents like alkaloids, volatile and essential oils, phenolic compounds, triterpenoids, saponins, phytosterols, tannins, flavanoids all possess, anti-inflammatory and antioxidant activity [41]. Leaves of plants have been reported to accumulate inulins, tannins and other alkaloids which may be responsible for their medicinal properties. Other studies reported that leaves are the most widely used parts of plant [42]. This may be because some plants contain many secondary metabolites which could have different pharmacological activities and consequently treat different diseases [43], [44], [45] or that leaves can be identified clearly and labeled for commercial trade. Our studies begin to address the complexity of pharmacologic activities present in traditional plant medicines used in Cameroon and help to further our understanding of mechanisms for action and why specific plants are used to treat individual diseases. According to World Health Organization, medicinal plants may be the best source for a variety of drugs. More than 75% of the total population in developing countries relies on traditional medicines based on plant products [1]. Cameroon is rich in the variety of medicinal plants that could help to fight disease like the HIV/AIDS, the malaria and tuberculosis. These diseases are concentrated in lower income countries where the health care access is difficult. The use of medicinal plants could constitute a reservoir of new molecules important for antifungal, antibacterial, antiviral, antioxidant and anti-inflammatory substances therapies and research on composition and mechanism of action will create better treatment standards and improve the value of traditional plants as sources of new medications.

## Conclusion

The findings of this study support the view that medicinal plants are promising sources of potential antioxidants and anti-inflammatory agents that may be effective for therapy of human diseases. Studying the ethanol soluble molecules revealed a rich variety of antioxidant and anti-inflammatory molecules mainly present in leaves. In the context of HIV/AIDS and other infections, research and development of novel and effective treatments from safe herbal medicines will improve the quality of life for persons in need. The results presented here should encourage the use of these plants for medicinal health and nutraceutical applications, due to their antioxidant and anti-inflammatory properties.
