# Correction: Antioxidant and Anti-Inflammatory Activities of Extracts from *Cassia alata, Eleusine indica, Eremomastax speciosa, Carica papaya* and *Polyscias fulva* Medicinal Plants Collected in Cameroon

**DOI:** 10.1371/journal.pone.0112573

**Published:** 2014-10-30

**Authors:** The PLOS ONE Staff

There is an error in the last sentence of the “Chemiluminescence Measurement of Plant Extracts Antioxidant Activity” section of the Materials and Methods. The correct sentence is: The data were reported as the percentage (%) of inhibition (I) of the CL (chemiluminescence) signal and calculated as follows:
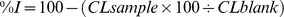
where ***CL*** **sample** is the chemiluminescence signal obtained for a sample in the presence of plant extracts and ***CL*** **blank** is the chemiluminescence signal obtained in the sample without plant extracts.
